# Puerperal Insanity

**Published:** 1861-08

**Authors:** 


### PUERPERAL INSANITY.

[We extract the concluding pages of a very valuable article, too lengthy for our space, in the July No. of the Am. Jour. of Insanity from the pen of Dr. J. H. Worthington.—*Ed.*]

The prevention of puerperal insanity will consist in placing the female under the most favorable hygienic influences which her circumstances will allow. Care should be taken to guard against every source of exhaustion. It ought to be remembered that the discharge from an extensive surface, like that of the interior of the uterus after parturition, cannot fail to reduce the strength of the patient. Her diet, therefore, while it is unstimulating should be nutricious. In her susceptible condition, she should be guarded from all excitement, such as unnecessary visits and conversation, and kept as much as possible out of the reach of anxiety and other painful emotions. Errors of regimen, such as exposure to cold, and indulgence in improper articles of diet, should be avoided. During inaction, if symptoms of anemia should be manifested the infant should at once be weaned, care being taken to avoid the distension of the breasts with the lacteal secretion, and the formation of abscesses. The return of menstruation should be carefully watched, and especial care taken to avoid every exposure until the function is re-established.

In puerperal as in ordinary insanity, so little is positively known of the anatomical lessions which characterize the disease, that in forming a judgment of its nature we are obliged to depend on our knowledge of its causes, and the circumstances under which it most commonly originates, rather than upon the structural changes it produces. The data furnished by morbid anatomy are almost entirely of a negative character; autopsies in the fatal cases having generally failed to show any positive evidence of inflammatory action. It is true that the recent researches of Dr. Calmeil have led him to conclude that acute delirium, which so frequently supervenes in fatal cases of puerperal insanity, is an inflammatory discolor of the cortical cerebral substance; yet he considers it an inflammation which is generally, if not always, met with in exhausted conditions of the system, and can not be treated by active, antiphlogistic measures. In regard to the nature of the affection, Dr. Macdonald remarks:

"When insanity occurs during the puerperal state we would expect to find the disease one of irritation rather than of inflammation; for it is admitted by all that the susceptibility of the female is never greater than during this period. She has been exhausted by uterogestation, while from the growth of the fœtus she has required more mourishment than usual; the irritability of her stomach has perhaps prevented her from using her accustomed quantity of food; she has been debarred from exercise in the open air, that preserver of life and health, and has been worn down by the doubts, and fears, and anxieties that are so apt to hang over the minds of women under these circumstances. In this state we would not look for inflammation; nevertheless we may sometimes meet with it, as we do with pneumonia in typhus, or after severe injuries or surgical operations, where there has been great loss of blood and strength. But it is not that active, vigorous inflammation which occurs in strong individuals. It is an inflammation which, judicious practitioners tell us, is often more successfully treated by stimulants in conjunction with other remedies. When it occurs during location we would expect to find a disease of debility, and we do find the mother pale, emaciated, reduced by suckling a large vigorous child, and by nights of watchfulness and anxiety for her offspring."

Various therapeutic measures have been proposed in the treatment of puerperal mania, among which may be mentioned local or general bloodletting, tartar emetic used as a contra-stimulant, warm baths, purgatives, narcotics, etc. I need not enter into any detail respecting the operation of these remedies. It will be sufficient to say, in regard to *all* depleting measures, that notwithstanding the appearance of energy and vital activity which may be supposed to be manifested by the quick pulse and great muscular strength, often exhibited by patients in a condition of extreme maniacal excitement, experience has proved, in too many instances, the injurious effects of all remedies of this description. In many cases in which, judging from the high grade of the delirium alone, we might be disposed to believe in the existence of inflammation, and consequently to resort to depletion, pain in the head, increased heat of the scalp, general fever, and other signs of inflammation are absent, and so far from being of service, any active depletion will then invariably render the condition of the patient more unfavorable. If, in an exceptional case, or in one in which the diagnosis is doubtful, the pulse should be found full and strong, and the condition of the patient appear to require depletion, it should be resorted to with the utmost caution, and its effects should be carefully watched.

In the commencement of an attack of puerperal mania, it will generally be necessary to have the patient placed in a room partially shaded, which shall be kept as quiet as possible, and into which no one shall be allowed to enter except the necessary attendants and nurses. A warm bath, continued for one, two or three hours in the evening, will tend to allay nervous irritation, and promote sleep. The bowels should be regulated by simple laxatives, and as there will generally be found a vitiated condition of the mucous membranes, evidenced by a furred tongue and offensive breath, laxatives should be continued daily with small portions of blue mass, until the tongue shows signs of improvement. Some gentle narcotic, of which a combination of asafœtida, hyoscyamus and camphor is a good example, should be given every three or four hours. The diet should be unstimulating but nutritious, and may advantageously consist in great part of new milk, given as frequently as the patient can be induced to take it, or as the stomach will bear it. If the acceleration of the pulse, the dry tongue, and the increase of maniacal excitement should indicate the supervention of acute delirium, the same treatment should be continued, but in these cases great difficulty will generally be experienced in controlling the movements of the patient, and in administering the necessary food and medicine. In this state there is a strong tendency to prostration, under the combined influence of the excessive muscular efforts of the patient, her almost total deprivation of sleep, and obstinate refusal of food. It is absolutely necessary, under these circumstances, to restrain the motions of the patient, by confining her with a bed-strap, by which means her strength will be husbanded, and the entire management of the case will be rendered more easy, and at the same time effectual. It is very important in these cases that the physician should have entire control of the bodily movements of the patient, in order that he may be able to apply warmth to the extremities if it should be needed, and also that he may have it in his power to administer food and drinks in such quantities as are necessary to support the strength of the patient. Under these cardinal points are attended to, but little good can be expected from the exhibition of medicines. In many cases of acute delirium the patients refuse food with such obstinacy that it is impossible, by any ordinary means, to give it in sufficient quantity. The œsophagus tube, which should be small enough to pass through the nostril, must then be resorted to, and a pint or more of milk, beef-tea, or other liquid nourishment, be injected into the stomach twice daily. If there should be increased heat of the scalp, it may be sometimes removed by warmth to the extremities, or by a dose of laxative medicine, or, if these means fail, by the application of cold to the head. Mustard pediluvia in the evening are always of service, and blisters to the extremities have been found useful as a derivative. It sometimes happens that, with considerable mental anxiety and distress, evidences of cerebral expression will become apparent; the patient is conscious of every thing about her, but appears to labor under difficulty in collecting her thoughts, and in recalling the occurrences of the day. In attempting to do so her mind becomes confused, and the effort fails of its object. With these symptoms of oppression and tendency to coma, I have found, in the absence of fever, the application of a large blister covering the entire scalp to be followed by a decided improvement in the cerebral disorder, and relief to all the symptoms.

